# Development of a suicide index model in general adolescents using the South Korea 2012–2016 national representative survey data

**DOI:** 10.1038/s41598-019-38886-z

**Published:** 2019-02-12

**Authors:** Jinhee Lee, Ho Jang, Jongkoo Kim, Seongho Min

**Affiliations:** 1 Department of Psychiatry, Yonsei University Wonju College of Medicine, Wonju, Korea; 2 Future Medicine division, Korea institute of Oriental Medicine, Daejeon, Korea; 3 Department of Family medicine, Yonsei University Wonju College of Medicine, Wonju, Korea

## Abstract

Suicide is a leading cause of death among adolescents and a major public health concern. Here we developed a risk stratification model for adolescent suicide attempts using sociodemographic characteristics, risk behaviours and psychological variables. Participants were 247,222 subjects in the Korea Youth Risk Behavior Web-based Survey (KYRBS). We developed a suicide index based on the suicide risk estimated in the generalized linear model and proposed the risk stratification model using the R language to measure the probability of suicide attempt among adolescents. Among the study population, the annual rate of suicide attempt was approximately 4%. The model provided good prediction for suicide attempt (AUC = 0.85). The important univariate risk factors for the outcome were dimensional measures of age, sex, breakfast consumption, experience of violence, sleep duration, perceived stress, feeling of sadness, current cigarette smoking, current alcohol drinking, perceived general health, perceived academic record, household economic status and living with biological or adoptive parents. Our suicide index model allowed the identification of adolescents who are at a high risk for suicide. This tool may promote the prevention of adolescent suicide and can be particularly useful in everyday settings where it is difficult to contact mental health professionals immediately.

## Introduction

Adolescent suicide is a major public health issue and accounts for an estimated 6% of all causes of death among young people worldwide^1^. In Korea, the rate of death due to suicide is significantly higher compared with that in other developed countries^2^. Moreover, the most common cause of death in Korean adolescents is suicide; however, among adolescents in other developed countries, road traffic accidents are the most common cause of death^3,4^. In fact, the rate of suicide attempts among Korean adolescents has continuously increased; in 2016, the suicide rate in individuals aged 12–18 years was 7.9 per 100,000, which was twice as that in 2001^5^.

Suicide attempt is the strongest known clinical predictor of completed suicide^6^ and is the main risk factor for continued suicidal behaviour and future death due to suicide in adolescents^7^. The ratio of completed suicide to suicide attempt is approximately 1:10^8^, and approximately 10–15% of those who attempted suicide finally died due to it^9^. Therefore, early detection of the adolescents who have attempted suicide and identifying them as a high risk group of suicide is extremely important. Because adolescents are more impulsive and emotionally unstable compared with adults, they are more likely to unexpectedly attempt suicide; this makes it difficult to detect adolescent suicide early^10^. Moreover, considering that many adolescents often hide or under report their mental health problems or disclose concerns on some parts of the situation only^11,12^, identifying their suicidal behaviours becomes more difficult. According to a large survey, 24–36% of adolescents reported having suicidal thoughts in the past year, but very few went on to seek further help for their problems^13^.

Several researches have demonstrated various risk factors for adolescent suicide; these include substance use, childhood abuse and parental psychopathology^14,15^. Other significant factors include problems in relationships with family or friends^16^, access to the means of self-harm^17^ and personality factors^18,19^. However, considering the complex mechanism of suicide occurrence, the individual application of each risk factor alone may be biased or inaccurate to predict and manage the suicidal behaviours of patients in the real clinical setting. Therefore, the overall combined effect of these risk factors needs to be considered in order for these to be applicable to the actual clinical setting or public health site.

A recent large-sample study on adults suggested a prediction model for suicide death using Cox regression, support vector machine and deep learning^20^. However, there remains a great lack of research on the process of selecting risk factors, the respective impact of each factor and the overall impact of these factors by a complex mechanism. Furthermore, there is no predictive model for suicide attempt in adolescents and most of the previous studies on adolescent suicide attempt focused largely on psychiatric patients or biased samples. The present study proposed a risk stratification model for adolescent suicide attempts using community-based national representative samples to collect data, including sociodemographic variables, risk behaviours and psychological variables that are readily available in everyday life.

## Methods

### Study population and source of data

The present study was performed using data obtained from the 2012–2016 Korea Youth Risk Behaviour Web-based Survey (KYRBS), which was established in 2005 by the Centers for Disease Control and Prevention in South Korea and is an ongoing annual nationwide cross-sectional survey that uses a stratified multi-stage cluster strategy among middle- and high-school students^21^. The KYRBS, which assessed the prevalence of health risk behaviours among adolescents, contains more than 100 questions that are divided into multiple sections on sociodemographic characteristics, health-related behaviours and mental and physical health^22^. After the survey had been fully explained, the participants were provided written informed consent to participate in the survey and provided with identification numbers. All participants were guaranteed anonymity before being asked to complete an online self-reported questionnaire and completed the anonymous self-administered web-based questionnaire in a school computer room. All data used in this study have been completely anonymized before accession and were analyzed anonymously. The KYRBWS was approved by the Institutional Review Board of the Korea Centers for Disease Control and Prevention. (Statistics Korea, approval No. 11758). All methods in the study were carried out with relevant guidelines and regulations.

The samples in this study were derived from the following datasets: 8th KYRBWS 2012 (N = 76,980, 96.4% response rate); 9th KYRBWS 2013 (N = 75,149, 96.4% response rate); 10th KYRBWS 2014 (N = 75,149, 97.2% response rate); 11th KYRBWS 2015 (N = 70,362, 96.7% response rate) and 12th KYRBWS 2016 (N = 67,983, 96.4% response rate). We excluded participants with incomplete information on self-questionnaires. Finally, we included 247,222 subjects for the present analysis and for the development of the suicide index model.

### Procedures and statistical analysis

Considering the most important risk factor correlated with adolescent suicide is a previous suicide attempt^14^, we set suicide attempt as an outcome variable of the suicide index model. Suicide attempt as the outcome variable was defined as a positive response to the question ‘In the past year, have you ever attempted suicide?’. The subjects were asked to respond with either (1) no, I never attempted suicide or (2) yes, I have attempted suicide.

For the suicide index model, we first used a logistic regression model with several predictor variables before selecting the independent variables by two processes. As a first step, a psychiatrist outlined the candidate variables that could influence suicide attempt. Through this first step, we screened from a literature-based search the following covariates that were previously demonstrated to be related with the risk of suicide attempt: age, sex, breakfast consumption, experience of violence, sleep duration, perceived stress, feelings of sadness, current cigarette smoking, current alcohol drinking, chronic allergic disease, perceived health status, perceived academic record, residential area, household economic status, paternal/maternal education level and living with biological or adoptive parent. The detailed information about the references in the literature-based search for variables was described in Supplementary Table 1. As a second step, a computer scientist determined the input variables for the final suicide index model through a statistical method. In this step, we performed binary logistic regression between the candidate variables and suicide attempt in order to select the covariates that were significantly related with the suicide attempt. These accepted variables from binary analysis were re-evaluated by backward stepwise logistic regression.

Statistical analysis was performed using the Statistical Package for the Social Science (SPSS ver. 20.0; IBM Corp., Armonk, NY). Data distribution was assessed to be normal, thereby, allowing the use of the Student’s t-test, which is a parametric test, for continuous variables, such as age and sleep duration. For categorical variables, the Chi-square test was used to compare the frequencies between groups. *P* < 0.05 was considered statistically significant.

We determined the 2012–2015 KYRBS and 2016 KYRBS data as the training and validation datasets, respectively. We determined a generalised linear model (GLM) for classification and to come up with the probability of suicide attempt among adolescents. The final GLM model was constructed from the training dataset and was validated through the validation dataset.

We measured the area under the receiver operating characteristic (ROC) curve and the F-measure of the test dataset to assess the performance of the final model, which was graphically illustrated as the ROC curve and F-score plot, respectively. R language (R packages, ver. 3.4.1) was used for constructing the GLM model and iteratively reweighted least squares was used for optimising the parameters.

## Results

The general characteristics of the training and testing datasets are presented in Tables 1 and 2, respectively. The mean age was younger in the suicide group than in the non-suicide group in both the training dataset (14.80 years vs. 15.15 years; *P* < 0.001) and testing dataset (14.95 years vs. 15.19 years; *P* < 0.001). In both the training and testing datasets, adolescent women were more likely to attempt suicide than did men; less consumption of breakfast, more experience of violence, less sleep duration, more perceived stress, more cigarette smoking and more alcohol drinking were significantly observed in the suicide group, compared with those in the non-suicide group. In both the training and testing datasets, compared with the non-suicide group, the suicide group had more number of participants with ≥2 chronic allergic diseases, who perceived health status as poor and academic record as low, who lived in the rural area, with low household economic status and who lived with one parent or others who were not their parents.

**Table 1:** Baseline characteristics of the training dataset.

	Training dataset (2013–16 KYRBS) N = 247,222		*P*-value
	Non-suicide attempt	Suicide attempt	
	N = 240,166	N = 7,056	
Age, years	15.15 ± 0.004	14.80 ± 0.021	<0.001
Sex (female), n	120,717 (50.3)	4,570 (64.8)	<0.001
Breakfast consumption, n	209,050 (87.0)	5,840 (82.8)	<0.001
Experience of violence, n	4,590 (1.9)	785 (11.1)	<0.001
Sleep duration, hours	6.21 ± 0.003	5.94 ± 0.019	<0.001
Perceived stress, n	91,316 (38.0)	5,470 (77.5)	<0.001
Feelings of sadness, n	63,278 (26.3)	5,623 (79.7)	<0.001
Current cigarette smoking, n	19,497 (8.1)	1,453 (20.6)	<0.001
Current alcohol drinking, n	40,254 (16.8)	2,193 (31.1)	<0.001
Chronic allergic diseases^a^ ≥2	37,910 (15.8)	1,389 (19.7)	<0.001
Perceived health status, n			<0.001
Excellent/good	171,141 (71.3)	3,537 (50.1)	
Average	54,310 (22.6)	2,236 (31.7)	
Fair/poor	14,715 (6.1)	1,283 (18.2)	
Perceived academic record			<0.001
High	97,085 (40.4)	2,206 (31.3)	
Middle	67,629 (28.2)	1,633 (23.1)	
Low	75,452 (31.4)	3,217 (45.6)	
Residential area			0.041
Rural	21,546 (9.0)	703 (10.0)	
Urban	109,436 (45.6)	3,187 (45.2)	
Metropolitan	109,184 (45.5)	3,166 (44.9)	
Household economic status			<0.001
High	85,321 (35.5)	2,290 (32.5)	
Middle	112,435 (46.8)	2,746 (38.9)	
Low	42,410 (17.7)	2,020 (28.6)	
Maternal education level(college), n	112,872 (47.0)	3,151 (44.7)	<0.001
Paternal education level(college), n	133,637 (55.6)	3,656 (51.8)	<0.001
Living with biological or adoptive parent			<0.001
Living with both parents	201,800 (84.0)	5,342 (75.7)	
Living with one parent	31,956 (13.3)	1,339 (19.0)	
Other	6,410 (2.7)	375 (5.3)	

Continuous variables are presented as mean ± standard deviation, and categorical variables are presented as number and ratio.

^a^Chronic allergic diseases include allergic rhinitis, allergic dermatitis, and asthma.

**Table 2:** Baseline characteristics of the training and testing datasets.

	Testing dataset (2017 KYRBS) N = 42,814		*P*-value
	Non-suicide attempt	Suicide attempt	
	N = 41,844	N = 970	
Age, years	15.19 ± 0.008	14.95 ± 0.057	<0.001
Sex (female), n	21,240 (50.8)	636 (65.6)	<0.001
Breakfast consumption, n	35,034 (83.7)	758 (78.1)	<0.001
Experience of violence, n	751 (1.8)	106 (10.9)	<0.001
Sleep duration, hours	6.13 ± 0.007	5.83 ± 0.051	<0.001
Perceived stress, n	15,349 (36.7)	732 (75.5)	<0.001
Feelings of sadness, n	10,114 (24.2)	764 (78.8)	<0.001
Current cigarette smoking, n	2,382 (5.7)	146 (15.1)	<0.001
Current alcohol drinking, n	6,645 (15.9)	289 (29.8)	<0.001
Chronic allergic diseases^a^ ≥2	7,726 (17.4)	192 (19.8)	<0.001
Perceived health status, n			<0.001
Excellent/good	30,591 (73.1)	464 (47.8)	
Average	8,722 (20.8)	319 (32.9)	
Fair/poor	2,531 (6.0)	187 (19.3)	
Perceived academic record			<0.001
High	18,156 (43.4)	337 (34.7)	
Middle	11,966 (28.6)	243 (25.1)	
Low	11,722 (28.0)	390 (40.2)	
Residential area			0.563
Rural	3,016 (7.2)	62 (6.4)	
Urban	19,689 (47.1)	461 (47.5)	
Metropolitan	19,139 (45.7)	447 (46.1)	
Household economic status			<0.001
High	17,768 (42.5)	382 (39.4)	
Middle	18,594 (44.4)	374 (38.6)	
Low	5,482 (13.1)	214 (22.1)	
Maternal education level(college), n	23,877 (59.0)	506 (55.7)	0.013
Paternal education level(college), n	26,096 (62.4)	570 (58.8)	<0.001
Living with biological or adoptive parent			<0.001
Living with both parents	35,306 (84.4)	728 (75.1)	
Living with one parent	5,509 (13.2)	195 (20.1)	
Other	1,029 (2.5)	47 (4.8)	

Continuous variables are presented as mean ± standard deviation, and categorical variables are presented as number and ratio.

^a^Chronic allergic diseases include allergic rhinitis, allergic dermatitis, and asthma.

Table 2 shows the results of univariate and multivariate logistic regression analyses of the covariates for suicide attempt. On univariate logistic regression, all the selected variables that were demonstrated in literature to be related with the suicide had significant results and were included in the backward stepwise logistic regression (Table 3). In model 1, the number of chronic allergic diseases, residential area and parental education level had non-significant associations with suicide attempt; the other variables were entered into the next stepwise logistic regression for model 2. Finally, 13 variables were determined as input features for the suicide risk stratification model (Table 3).

**Table 3:** Backward-stepwise logistic regression for selecting the input variables.

	Univariate	Multivariate (Model 1)	Multivariate (Model 2)
	OR (95% CI)	OR (95% CI)	OR (95% CI)
Age (years)	0.890 (0.878–0.902)	0.755 (0.741–0.768)	0.759 (0.747–0.772)
Sex (female)	1.819 (1.731–1.911)	1.461 (1.379–1.549)	1.490 (1.410–1.574)
Breakfast consumption	0.715 (0.671–0.761)	0.907 (0.844–0.975)	0.894 (0.836–0.956)
Experience of violence	6.425 (5.932–6.958)	3.468 (3.149–3.819)	3.528 (3.227–3.857)
Sleep duration (hours)	0.875 (0.861–0.890)	0.914 (0.895–0.933)	0.917 (0.900–0.935)
Perceived stress	5.622 (5.313–5.949)	2.434 (2.280–2.598)	2.392 (2.248–2.545)
Feelings of sadness	10.969 (10.344–11.632)	6.231 (5.834–6.656)	6.294 (5.911–6.702)
Current cigarette smoking	2.935 (2.765–3.115)	1.984 (1.828–2.152)	2.015 (1.869–2.174)
Current alcohol drinking	2.240 (2.127–2.358)	1.546 (1.445–1.655)	1.506 (1.413–1.605)
Chronic allergic diseases ≥2	1.308 (1.232–1.388)	1.037 (0.971–1.108)	—
Perceived health status (poor)	2.042 (1.979–2.108)	1.386 (1.336–1.439)	1.385 (1.337–1.434)
Perceived academic record (good)	1.387 (1.348–1.426)	1.103 (1.068–1.139)	1.092 (1.059–1.126)
Residential area (metropolitan)	0.962 (0.928–0.998)	1.002 (0.961–1.044)	—
Household economic status (low income)	1.315 (1.273–1.359)	1.048 (1.008–1.089)	1.038 (1.002–1.076)
Maternal education level (college)	0.940 (0.900–0.981)	1.041 (0.984–1.101)	—
Paternal education level (college)	0.890 (0.854–0.929)	0.996 (0.942–1.053)	—
Living with biological or adoptive parent (No living parents)	1.526 (1.463–1.592)	1.139 (1.068–1.214)	1.198 (1.143–1.255)

Abbreviations: OR, odds ratio; CI, confidence interval.

The coefficients of the generalised linear model that included the 13 input features are presented in Table 4. The top three coefficients that were positively related with an increased suicide attempt rate were feeling of sadness, experience of violence and perceived stress. In addition, the bottom three coefficients that were negatively related with suicide attempt were age, breakfast consumption and sleep duration.

**Table 4:** Regression coefficients of the final model for predicting suicide attempt.

	Score	Β	SE	*P*-value
Age	Years	−0.275	0.009	<0.001
Sex	Men = 1	0.399	0.399	<0.001
	Women = 2			
Breakfast consumption	Yes = 1	−0.112	0.034	0.001
	No = 0			
Experience of violence	Yes = 1	1.261	0.045	<0.001
	No = 0			
Sleep duration	Hours	−0.086	0.010	<0.001
Perceived stress	Yes = 1	0.872	0.032	<0.001
	No = 0			
Feelings of sadness	Yes = 1	1.840	0.032	<0.001
	No = 0			
Current cigarette smoking	Yes = 1	0.701	0.039	<0.001
	No = 0			
Current alcohol drinking	Yes = 1	0.409	0.033	<0.001
	No = 0			
Perceived general health	Good = 1	0.326	0.018	<0.001
	Fair = 2			
	Bad = 3			
Perceived academic record	High = 1	0.088	0.016	<0.001
	Middle = 2			
	Low = 3			
Household economic status	High income = 1	0.038	0.018	0.038
	Middle income = 2			
	Low income = 3			
Living with biological or adoptive parent	Both Parents = 1	0.180	0.024	<0.001
	One Parent = 2			
	Others = 3			
Constants		−6.648	0.209	

Abbreviation: SE, standard error.

As suggested by previous studies^23,24^, the suicide risk concentration was analyzed by the tier of predicted probability and OR between suicidal death and the tier of the calculated probabilities (Table 5). In group 1, the expected and observed suicide attempt ratios were 0.5% and 7.0%, respectively. Considering the bottom 10% of the predicted probability as the reference group, the top 0.5% of the predicted probability had an OR of 400. Further, the percentages of suicidal death gradually increased to the top of each group, except for groups 2 and 3. This result indicated the feasibility of risk-stratified preventive interventions using the tiers of suicidal death predicted probability calculated by our model.

**Table 5:** Suicide risk concentration by tier of predicted probability calculated by the generalised linear model (n = 42,814, testing dataset).

Tier of predicted probability, %	Suicide attempt, n^a^	Overall, n^a^	OR (95% CI)
Group 1: 0.0–0.5	68 (7.0)	214 (0.5)	399.52 (158.74–1005.6)
Group 2: 0.5–1	30 (3.1)	214 (0.5)	139.86 (53.65–364.61)
Group 3: 1–2	86 (8.8)	428 (1.0)	215.70 (86.70–535.0)
Group 4: 2–5	138 (14.3)	1284 (3.0)	103.30 (42.22–252.71)
Group 5: 5–10	154 (15.9)	2140 (5.0)	66.52 (27.26–162.34)
Group 6: 10–50	442 (45.6)	17120 (40.0)	22.73 (9.41–54.92)
Group 7: 50–90	47(4.8)	17120 (40.0)	2.361 (0.939–5.941)
Group 8: 90–100	5 (0.5)	4294 (10.0)	1.000 (Reference)
Total	970 (100.0)	42814 (100.0)	—

^a^Categorical variables are presented as number (percent).

Abbreviation: OR, odds ratio; CI, confidence interval.

**Figure 1:**
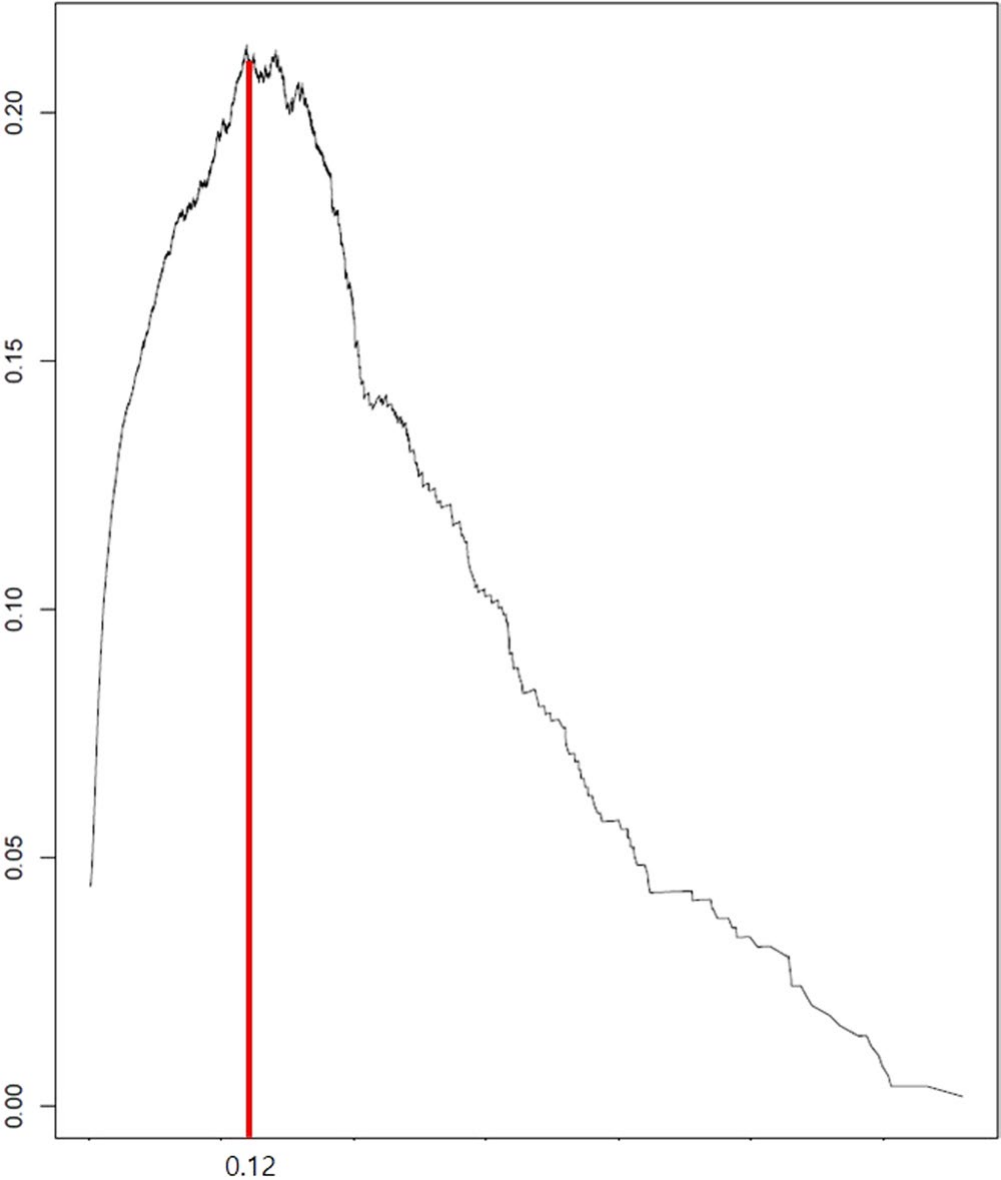
F-score curve of test dataset (KYRBS 2016). The suicide model generated the maximum F-score of 0.23 for participants attempting suicide.

From these parameters, we obtained the suicide index (Appendix), which was a score between 0 and 1 to indicate the probability of a suicide attempt. Assessment of the performance of this model from the testing dataset yielded an area under the receiver operating curve (AUC) of 0.85 (Supplementary Fig. 1). Furthermore, the cut-off for the suicide index was determined as 0.12 or 12% based on the maximum value of the f-measure (Fig. 1).

## Discussion

Using the annual national representative data from the 2013 to 2017 KYRBWS, we proposed the use of a suicide index as a predictive model for adolescent suicide attempt. We calculated the combined effects of the risk factors, rather than simply measuring the separate risk for suicide attempt with each factor. This model provided relevant discrimination between those who had suicide attempt and those who did not over the past year.

The AUC of 0.85 obtained for the suicide index in this study can be compared with that of the other prediction models that were previously used in the field of psychiatry [e.g. suicide deaths of adults (AUC 0.68), new-onset psychosis (AUC 0.79) and bipolar disorder (AUC 0.76)]^20,25,26^. The 13 simple predictors are easily to obtain in a primary care or school health setting. Therefore, this simple risk calculator can be easily used every day and can provide a tool for not only clinicians, but also for teachers, family members and friends to rapidly identify those who have risks for suicidal behaviour. Such tools can be particularly useful in settings, such as homes and schools, where it is difficult to contact mental health professionals immediately.

Although risk calculators have been widely used in other medical fields, development of prediction models in psychiatry had some limitations. A number of predictive models have been identified in a recent review article, but most of these models were for depression and psychosis^27^. In addition, a small of number of suicide prediction studies were conducted based on mental pathology, such as depression and behavioural disorders^2,20^. On the other hand, this study highlighted the elaborate logical developments, and the generalisability of this model can precisely explain the complex mechanism of adolescent suicides.

In this study, combination of the top three suicide attempt-related variables (i.e. feeling of sadness, experience of violence and perceived stress) generated an AUC of 0.82. Addition of the other variables selected from literature and by statistical validation improved the AUC to 0.85. These results indicated that public information, which can be readily obtained from questionnaires but are usually ignored, could help predict the risk for suicidal behaviours in adolescents.

Depressive mood is one of the most important risk factors of adolescent suicide. A study on 1,176 non-suicidal subjects and 109 subjects with previous suicide attempts or suicidal ideation, showed that depressive disorder was significantly associated with the risk of suicide (odds ratio, 11.4)^28^. The present study investigated the risk of suicide attempt by replacing the diagnosis of depression with information on feelings of sadness, which can be more readily collected in everyday life, and generated a similar risk (odds ratio, 6.294). Further, in the final model of this study, the single variable of sadness feelings generated an AUC of 0.773, indicating that, by itself, this variable was useful enough to determine the risk of suicidal behavior.

Previous studies have demonstrated that compared with those who had not, adolescents who had been victims of violence during the past year were more likely to experience negative mental health outcomes, including suicidal ideation (odds ratio, 5.41)^29^. However, there were substantial differences among the studies in terms of response rate, design, confounders that were controlled for and the use of self-reports of victim of violence^30^. In the suicide index model in this study, the association of the variable victim of violence with suicide attempt (odds ratio, 3.52 and AUC, 0.63) further confirmed that this variable is an important risk factor for adolescent suicide. Consistent with a previous study^31^, the current study also demonstrated that subjects who had a high stress level had a higher probability of suicide attempt, compared with those who had low stress level (odds ratio, 2.392).

The main task of a risk prediction index model is to identify an optimal set of risk factors that best predicts the outcome. Many of the variables, such as age, sex and substance use, which predicted adolescent suicide attempt in this study were also reported by previous studies meta-analysis and review articles. However, some factors, including chronic allergic disease, residential area and paternal education level, which were found on literature to be risk factors for suicide attempt were not included in the suicide index, because these variables were not associated with the risk for suicide after adjusting the covariates.

Considering that a past suicide attempt is one of the most important risk factors for future suicides, our model could be used to screen for high-risk groups. It has been suggested that some suicide attempts may be preventable, if the problem of under-treatment can be overcome by the sensitive insights of health professionals and the public^32^. In this respect, building an individualised risk calculator may be an important practical tool for clinicians and public health professionals to assess the suicide risk in adolescents who had a suicide attempt in the past year and to plan further evaluations and necessary early interventions. Changes in the risk score can be monitored over time to provide a risk trajectory for suicidal adolescents and to evaluate the effectiveness of an intervention program. In future studies, we recommend development of an adjunctive risk calculator based on this algorithm using web-based, smartphone apps; nomograms or score chart^33^.

The key strength of the model generated in this study, which was based on a large national representative sample, was that it can predict the possibility of a suicide attempt using basic characteristics and demographic factors. The participants of this study had a very high response rate and had almost no missing data; these may have been due to the systematic support of national organizations and the technical advantages of online survey methods^34^. National surveillance systems for monitoring adolescent health risk behaviours have been implemented in many countries, and the reliability of the KYRBWS questionnaire had been validated for over time in a number of previous studies^35^. Moreover, we increased the predicting power and maximised the statistical value by the high performance of the risk stratification model and repeating validation. This study included participants from a general adolescent population, rather than from clinical patients; therefore, the results of this study could be universally applicable to the general adolescent population. The results of this study provided a practical tool that consists of a simple input of variables that can be easily obtained from daily life of the people around.

The present findings should be considered in light of some limitations. First, we used a cross-sectional design and the risk for future suicide was estimated indirectly from a past suicide attempt. Future works should investigate the risk for suicide attempt using longitudinally designed data and make a risk calculator that can directly predict future suicide attempts in adolescents. Second, our risk model was purposefully derived from a general population of adolescents, and the study lacked information on other potential covariates, such as clinical diagnosis, psychiatric symptoms and psychiatric/suicide family history, which were not available in the KYRBWS data. Third, although we had an adequate number of participants to build a risk model to predict suicide attempts, this study did not include data on adolescents outside of the school, which accounts for approximately 1.8% of those aged 12–17 years in Korea. Therefore, there may be limitations in applying the present risk models to all adolescents in the society. Fourth, there is a possibility that those respondents would be answering questions about their mental state and other characteristics after a suicide attempt. Therefore, the indicated level of psychological distress might partly result from the suicide attempt, rather than be a contributing factor. Further research is required to exclude the possibility of increasing the statistical association between such covariates and the dependent variables, further clearing the causality. Such research could involve prospective models, which are beyond the data and design of the current study. In a similar context, we hope the present study may be a cornerstone for further extended research exploring several types of potentially relevant covariates, such as clinical records, as previously performed in studies^20^.

To our best knowledge, this was the first study that used circular logic based on a large sample to create a prediction model for adolescent suicide attempt. We built our risk stratification model using the results from a recent meta-analysis^15^ and found that the combination of basic characteristics in daily life provided clinically relevant discrimination between those who had suicide attempts in the past year and those who have not. Replication of these findings and longitudinal research might be warranted, in order for the risk calculator to be used confidently by clinicians, teachers, friends and family members. The clinical application of the model presented in this study includes the development of websites or applications that can apply weights to this risk calculator. Furthermore, the provision of appropriate guidelines for screened suicide risk groups is needed for further studies. Nevertheless, this risk calculator can be a practical tool for assessing the risk for suicidal behaviour and for early interventions in adolescents with high suicidal risk.

## Case Study

### Suicidal behaviour classification and an example from the GLM model

The following formula represents the GLM model:

$$Risk=\sum _{i=1}^{m}\,{w}_{i}{x}_{i}+{w}_{0}$$

$$\mu =f\,(Risk)=\frac{1}{1+\exp \,(\,-\,Risk)}$$

where μ which indicates the mean of the distribution of the suicide index; *Risk* represents the linear predictor that is a weighted sum of the covariates (*x*_*i*_); and *f* represents an activation function, the inverse of which is the link function showing the relationship between the linear predictor and the suicide index. In an example case (Table 5), the risk of a suicidal attempt based on the GLM model is calculated, as follows:

**Table:** 

**Example case**		
**Variables**	**Parameters** ***w***_***i***_	**Values** ***x***_***i***_ **(Score)**
Age	−0.275	15
Sex	0.399	Female (2)
Breakfast consumption	−0.112	Having breakfast (1)
Experience of violence	1.261	Having experience (1)
Sleep duration	−0.086	6 hours
Perceived stress	0.872	Perceiving stress (1)
Feeling of sadness	1.840	Feeling of sadness(1)
Current cigarette smoking	0.701	Having experience (1)
Current alcohol drinking	0.409	Having experience (1)
Perceived health status	0.326	Fair/Poor (3)
Perceived academic record	0.088	Low (3)
Household economic status	0.038	Low (3)
Living with biological or adoptive parent	0.180	Living with others (3)
Bias	−6.648	

$$\begin{array}{rcl}a & = & \sum _{i=1}^{m}\,{w}_{i}{x}_{i}+{w}_{0}\\  & = & (\,-\,0.275)\times 15+0.399\times 2+(\,-\,0.112)\times 1+(1.261)\times 1\\  &  & +\,(\,-\,0.086)\times 6+0.872\times 1+1.840\times 1+0.701\times 1\\  &  & +\,0.409\times 1+0.326\times 3+0.088\times 3+0.038\times 3\\  &  & +\,0.018\times 3-6.648\\  & = & -3.624\end{array}$$

$$\mu =f\,(a)=\frac{1}{1+\exp \,(\,-\,3.624)}=0.025983\,\fallingdotseq \,2.6 \% $$

The suggested probability of the person’s suicide was reported as 2.6, which belongs to the high-risk group (μ > 0.12).

## Supplementary information

Supplementary Information

## Data Availability

The authors used data from the 12th Korea Youth Risk Behavior Web- based Survey (KYRBS 2016), co-administered by the Korean Ministry of Education, the Korean Ministry of Health and Welfare, and the Korean Centers for Disease Control and Prevention. The KYRBS dataset is publicly available via http://yhs.cdc.go.kr. Access to the dataset requires a simple application process via the official website. They require researchers to use the dataset only upon gaining access via the website. Each individual researcher who wants to use the KYRBS dataset must complete the application process.
