# Supplementary material for: Development of a suicide index model in general adolescents using the South Korea 2012–2016 national representative survey data
Source: Sci Rep. 2019 Feb 12;9:1846. doi: 10.1038/s41598-019-38886-z (PMC6372692; doi:10.1038/s41598-019-38886-z)
Supplement: Supplementary file 1 — Supplementary Information [file 41598_2019_38886_MOESM1_ESM.docx]

**Supplementary Information**

**Development of a suicide index model in general adolescents using the South Korea 2012–2016 national representative survey data**

Jinhee Lee^1^, Ho Jang^2^, Jongkoo Kim^3*^, and Seongho Min^1**^

^1^Department of Psychiatry, Yonsei University Wonju College of Medicine, Wonju, Korea

^2^Future Medicine division, Korea institute of Oriental Medicine, Daejeon, Korea

^3^Department of Family medicine, Yonsei University Wonju College of Medicine, Wonju, Korea

****Corresponding Author**

Seongho Min, MD, PhD

Professor & Chairperson, Department of Psychiatry, Yonsei University Wonju College of Medicine

20 Ilsan-ro Wonju, 26426, Republic of Korea

Tel. No.: +82-33-741-1260

E-mail address: [mchorock@yonsei.kr](mailto:mchorock@yonsei.kr)

***Co-corresponding Author**

Jongkoo Kim, MD, PhD

Professor & Chairperson, Department of Family medicine, Yonsei University Wonju College of Medicine

20 Ilsan-ro Wonju, 26426, Republic of Korea

Tel. No.: +82-33-741-1416

E-mail address: [kimjk214@yonsei.kr](mailto:kimjk214@yonsei.kr)

**Supplementary Table 1.** References in the literature-based search for variables

|  |  |  |  |
| --- | --- | --- | --- |
| Age, years | Self-harm and suicide in adolescents^1^  Suicide and suicidal behavior in adolescents^2^  Age-and sex-related risk factors for adolescent suicide^3^ | Hawton K  Bridge JA  Brent DA | 2012  2006  1999 |
| Sex (female), *n* | Self-harm and suicide in adolescents^1^  Suicide and suicidal behavior in adolescents^2^  Age-and sex-related risk factors for adolescent suicide^3^ | Hawton K  Bridge JA  Brent DA | 2012  2006  1999 |
| Breakfast consumption, *n* | Breakfast consumption and depressive mood: A focus on socioeconomic status^4^ | Lee SA | 2017 |
| Experience of violence, *n* | School bullying and suicidal risk in Korean middle-school students^5^  Suicidal ideation among adolescent school children, involvement in bully–victim problems, and perceived social support^6^  Bullying, depression, and suicidal ideation in Finnish adolescents: a school survey^7^ | Kim YS  Rigby K  Kaltiala-Heino R | 2005  1999  1999 |
| Sleep duration, hours | Sleep and adolescent suicidal behavior^8^ | Liu X | 2004 |
| Perceived stress, *n* | Adolescent suicide attempts: risks and protectors^9^ | Borowsky IW | 2001 |
| Feelings of sadness, *n* | Adolescent suicide attempts: risks and protectors^9^ | Borowsky IW | 2001 |
| Current cigarette smoking, *n* | Regular daily smoking among 14-year-old adolescents increases the subsequent risk for suicide: the Northern Finland 1966 Birth Cohort Study^10^  Smoking and suicidality among adolescent psychiatric patients^11^ | Riala K Mäkikyrö TH | 2007  2004 |
| Current alcohol drinking, *n* | Alcohol and adolescent suicide^12^  Predictors of adolescent suicide attempts: a nationally representative longitudinal study of Norwegian adolescents^13^ | Sher L  Wichstrøm L | 2005  2000 |
| Chronic allergic diseases ^a^≥2 | Depressive symptoms and suicide ideation in adolescent with allergic disease^14^  Allergy: a risk factor for suicide?^15^ | Seo J  Postolache TT | 2015  2008 |
| Perceived health status, *n* | Twelve-month prevalence and predictors of self-reported suicidal ideation and suicide attempt among Korean adolescents: a web-based nationwide survey^16^ | Kang E-H | 2015 |
| Excellent/good |  |  |  |
| Average |  |  |  |
| Fair/poor |  |  |  |
| Perceived academic record | Perceived academic performance, self-esteem, and locus of control as the indicators of need for assessment of adolescent suicide risk: implications for teachers^17^ | Martin G | 2005 |
| High |  |  |  |
| Middle |  |  |  |
| Low |  |  |  |
| Residential area | Suicide and suicidal behavior in adolescents^2^  Age-and sex-related risk factors for adolescent suicide^3^ | Bridge JA  Brent DA | 2006  1999 |
| Rural |  |  |  |
| Urban |  |  |  |
| Metropolitan |  |  |  |
| Household economic status | Self-harm and suicide in adolescents^1^  Suicide and suicidal behavior in adloescents^2^ | Hawton K  Bridge JA | 2012  2006 |
| High |  |  |  |
| Middle |  |  |  |
| Low |  |  |  |
| Maternal education level(college), *n* | Prevalence, correlates, and treatment of lifetime suicidal behavior among adolescents: results from the National Comorbidity Survey Replication Adolescent Supplement^18^ | Nock MK | 2013 |
| Paternal education level(college), *n* | Prevalence, correlates, and treatment of lifetime suicidal behavior among adolescents: results from the National Comorbidity Survey Replication Adolescent Supplement^18^ | Nock MK | 2013 |
| Living with biological or adoptive parent | Prevalence, correlates, and treatment of lifetime suicidal behavior among adolescents: results from the National Comorbidity Survey Replication Adolescent Supplement^18^ | Nock MK | 2013 |
| Living with both parents |  |  |  |
| Living with one parent |  |  |  |
| Other |  |  |  |
|  | | | |

**Supplementary Figure 1**


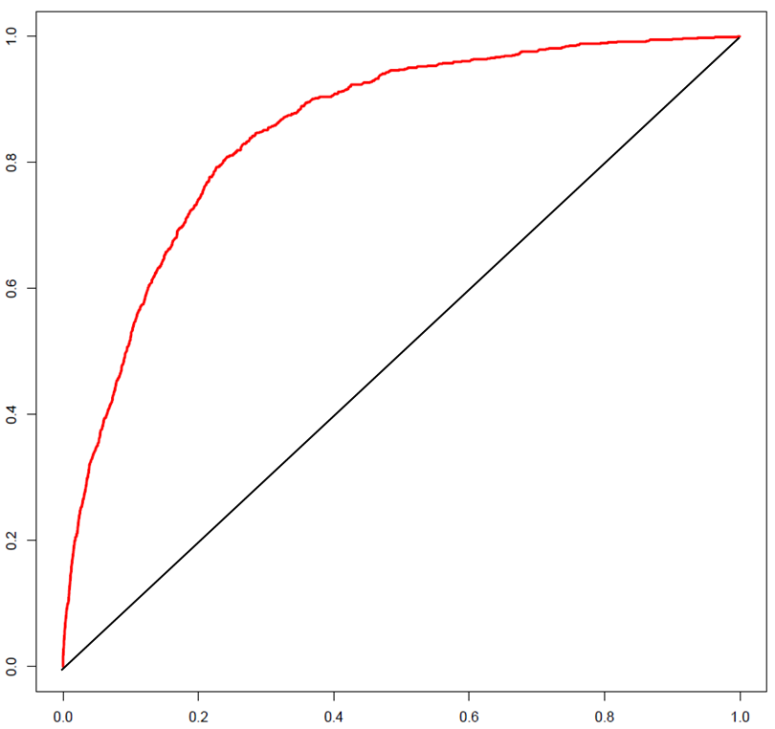


**Supplementary Figure 1.** Receiver operating characteristic (ROC) curve of the test dataset (KYRBS 2016)

The suicide model generated an area under the curve (AUC) of 0.85 for participants attempting suicide.

**References**

1. Hawton, K., Saunders, K. E. & O'Connor, R. C. Self-harm and suicide in adolescents. *Lancet* **379**, 2373–2382; https://doi.org/10.1016/S0140-6736(12)60322-5 (2012).

2. Bridge, J. A., Goldstein, T. R. & Brent, D. A. Adolescent suicide and suicidal behavior. *J. Child Psychol. Psychiatry* **47**, 372–394; https://doi.org/10.1111/j.1469-7610.2006.01615.x (2006).

3. Brent, D. A., Baugher, M., Bridge, J., Chen, T. & Chiappetta, L. Age-and sex-related risk factors for adolescent suicide. *J. Am. Acad. Child Adolesc. Psychiatry* **38**, 1497–1505; https://doi.org/10.1097/00004583-199912000-00010 (1999).

4. Lee, S. A. *et al.* Breakfast consumption and depressive mood: A focus on socioeconomic status. *Appetite* **114**, 313–319; https://doi.org/10.1016/j.appet.2017.04.007 (2017).

5. Kim, Y. S., Koh, Y. J. & Leventhal, B. School bullying and suicidal risk in Korean middle school students. *Pediatrics* **115**, 357–363; https://doi.org/10.1542/peds.2004-0902 (2005).

6. Rigby, K. & Slee, P. Suicidal ideation among adolescent school children, involvement in bully—victim problems, and perceived social support. *Suicide Life Threat. Behav.* **29**, 119–130; https://doi.org/10.1111/j.1943-278X.1999.tb01050.x (1999).

7. Kaltiala-Heino, R., Rimpelä, M., Marttunen, M., Rimpelä, A. & Rantanen, P. Bullying, depression, and suicidal ideation in Finnish adolescents: school survey. *BMJ* **319**, 348–351; https://doi.org/10.1136/bmj.319.7206.348 (1999).

8. Liu, X. Sleep and adolescent suicidal behavior. *Sleep* **27**, 1351–1358; https://doi.org/10.1093/sleep/27.7.1351 (2004).

9. Borowsky, I. W., Ireland, M. & Resnick, M. D. Adolescent suicide attempts: risks and protectors. *Pediatrics* **107**, 485–493; https://doi.org/10.1542/peds.107.3.485 (2001).

10. Riala, K. *et al.* Regular daily smoking among 14-year-old adolescents increases the subsequent risk for suicide: the Northern Finland 1966 Birth Cohort Study. *J. Clin. Psychiatry* **68**, 775–780; https://doi.org/10.4088/JCP.v68n0518 (2007).

11. Mäkikyrö, T. H. *et al.* Smoking and suicidality among adolescent psychiatric patients. *J. Adolesc. Health* **34**, 250–253; https://doi.org/10.1016/j.jadohealth.2003.06.008 (2004).

12. Sher, L. & Zalsman, G. Alcohol and adolescent suicide. *Int. J. Adolesc. Med. Health* **17**, 197–204; https://doi.org/10.1515/IJAMH.2005.17.3.197 (2005).

13. Wichstrøm, L. Predictors of adolescent suicide attempts: a nationally representative longitudinal study of Norwegian adolescents. *J. Am. Acad. Child Adolesc. Psychiatry* **39**, 603–610; https://doi.org/10.1097/00004583-200005000-00014 (2000).

14. Seo, J. Y. *et al.* Depressive symptoms and suicide ideation in adolescent with allergic disease. *J Korean Soc Biol Ther Psychiatry* **21**, 167–173 (2015).

15. Postolache, T. T., Komarow, H. & Tonelli, L. H. Allergy: a risk factor for suicide? *Curr. Treat. Options Neurol.* **10**, 363–376; https://doi.org/10.1007/s11940-008-0039-4 (2008).

16. Kang, E. H. *et al.* Twelve-month prevalence and predictors of self-reported suicidal ideation and suicide attempt among Korean adolescents in a web-based nationwide survey. *Aust. N. Z. J. Psychiatry* **49**, 47–53; https://doi.org/10.1177/0004867414540752 (2015).

17. Martin, G., Richardson, A. S., Bergen, H. A., Roeger, L. & Allison, S. Perceived academic performance, self-esteem and locus of control as indicators of need for assessment of adolescent suicide risk: implications for teachers. *J. Adolesc.* **28**, 75–87; https://doi.org/10.1016/j.adolescence.2004.04.005 (2005).

18. Nock, M. K. *et al.* Prevalence, correlates, and treatment of lifetime suicidal behavior among adolescents: results from the National Comorbidity Survey Replication Adolescent Supplement. *JAMA Psychiatry* **70**, 300–310; https://doi.org/10.1001/2013.jamapsychiatry.55 (2013).
